# Carotenoid Recovery from Tomato Processing By-Products through Green Chemistry

**DOI:** 10.3390/molecules27123771

**Published:** 2022-06-11

**Authors:** Katalin Szabo, Bernadette-Emőke Teleky, Floricuta Ranga, Ioana Roman, Hattab Khaoula, Emna Boudaya, Amina Ben Ltaief, Wael Aouani, Mangkorn Thiamrat, Dan Cristian Vodnar

**Affiliations:** 1 Faculty of Food Science and Technology, University of Agricultural Sciences and Veterinary Medicine Cluj-Napoca, Calea Manastur 3-5, 400372 Cluj-Napoca, Romania; katalin.szabo@usamvcluj.ro (K.S.); bernadette.teleky@usamvcluj.ro (B.-E.T.); floricutza_ro@yahoo.com (F.R.); ioana.roman@usamvcluj.ro (I.R.); 2 Institute of Biotechnology of Sfax, University of Sfax, 90 Ave Mohamed V, Tunis 1002, Tunisia; khhtb18@gmail.com (H.K.); boudayaemna45@gmail.com (E.B.); aminabenltaief96@gmail.com (A.B.L.); waelawani1996@gmail.com (W.A.); 3 School of Food Technology, Institute of Agricultural Technology, Suranaree University of Technology, Nakhon Ratchasima 30000, Thailand; mangkornthiamrat40@gmail.com

**Keywords:** bioavailability, circular economy, ethyl acetate, ethyl lactate, functional foods, lycopene, rheology

## Abstract

The recovery of bioactive compounds from agro-industry-derived by-products sustains circular economy principles by encouraging maximized recycling and minimized waste. Tomato processing by-products are abundant in carotenoids, which have several health-promoting properties, and their reintegration into functional food products represents a major interest for scientists and manufacturers. In the present study, carotenoids were recovered from tomato processing by-products based on the principles of green chemistry by using generally recognized as safe (GRAS) solvents, freeze-drying as pretreatment, and ultrasound in the recovery procedure. Spectrophotometric measurements and HPLC were used to identify and quantify total and individual carotenoids from the extracts. The highest values for lycopene (1324.89 µg/g dw) were obtained when ethyl lactate was applied as a solvent, followed by ethyl acetate with slightly smaller differences (1313.54 µg/g dw). The extracts obtained from freeze-dried samples presented significantly lower amounts of lycopene, indicating that carotenoids are highly susceptible to degradation during lyophilization. Flaxseed, grape seed, and hempseed oils were enriched with carotenoids and their rheological measurements showed favorable viscoelastic properties, especially hempseed and flaxseed oil, with viscosity under 50 mPa·s. Considering the results and the economic perspective of carotenoid recovery from tomato processing by-products, ethyl acetate is suitable, sustainable, and environmentally friendly for carotenoid extraction.

## 1. Introduction

Tomatoes (*Lycopersicon esculentum*) are the world’s second-most important vegetable crop and a key component of the Mediterranean diet, with a global production of over 186 million tons in 2020, according to the Food and Agriculture Organization (FAO, 2020, https://www.fao.org/faostat/en/#data/QCL, accessed date 25 April 2022). Tomato consumption is linked to a series of health benefits such as lower risk of inflammatory processes and chronic diseases, reduced risk of developing carcinogenesis, and the inhibition of low-density lipoprotein oxidation, which helps to lower blood cholesterol levels in humans [1,2].

According to recent studies, more than one-quarter of the global production of tomatoes is processed as tomato-based products such as tomato sauce, ketchup, and canned or crushed tomatoes, which are in high consumer demand. The industrial processing of tomatoes generates large quantities of by-products, namely tomato pomace composed of tomato peels, seeds, and small amounts of pulp, which are primarily used as livestock feed, compost or disposed [3]. The amount of waste ranges between 5.4 and 9.0 million tons yearly; however, precise statistics on this subject matter are difficult to collect [4]. The by-products can represent 5–30% of the main product and are a source of environmental discomfort caused by their high moisture content, which favors microorganism growth and further impacts global warming through methane emissions [5]. On the other hand, several scientific studies highlight the rich bioactive composition of tomato processing by-products and the possible reutilization of extracted phytochemicals such as carotenoids, phenolic compounds, dietary fiber, and polyunsaturated fatty acids [6,7,8]. These nutraceutical chemicals can be used as natural antioxidants, preservatives, colorants, and functional food ingredients along with pharmaceutical and cosmetic applications [9,10,11]; therefore, the search for new uses in industries is a critical trigger in moving beyond the image of “waste as a problem” to “waste as a resource” [12,13,14].

**Figure 1:**
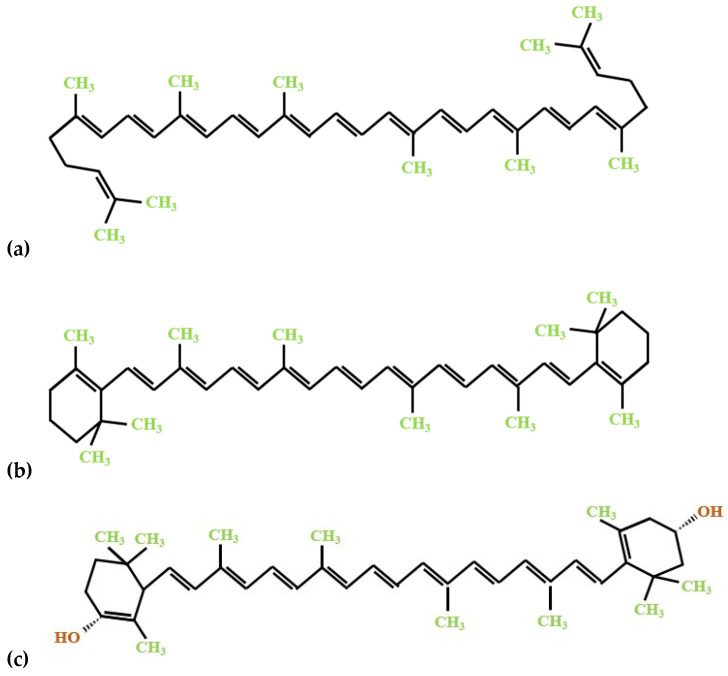
The chemical structure of the significant carotenoid constituents of tomato processing by-products: (**a**) lycopene; (**b**) β-carotene; (**c**) lutein.

The major bioactive constituents of tomato processing by-products are carotenoids, predominantly lycopene, followed by β-carotene and lutein (Figure 1). The human organism cannot produce these bioactive compounds; therefore, their uptake in the body relies on dietary sources such as carotenoid-rich foods and supplements. Given that carotenoids are fat-soluble pigments, specific fatty acids or vegetable lipids play an essential role in their bioavailability, and are involved in regulating the oxidative mechanism at the same time [15,16,17].

Lycopene is a bright red pigment that accumulates in tomato peels at concentrations approximately 5-foldhigher than in tomato seeds [18] and tomato pulp [19] and is the primary carotenoid found in tomatoes. Due to its superior antioxidant activity, lycopene has been found to significantly benefit human health, reducing the risk of several non-communicable diseases and other degenerative disorders mediated by free radical reactions [20]. Therefore, lycopene has been proposed and is already used in a wide range of industrial applications as a food supplement or as a functional ingredient in new food products [4]. This large number of possible applications of lycopene as a high-added-value product, combined with its abundance in tomato peels and with the growing consumer demand for natural food additives, underpins the great interest of researchers and manufacturers in the recovery of lycopene from tomato processing by-products discarded mostly from the peeling operation [4,21].

**Figure 2:**
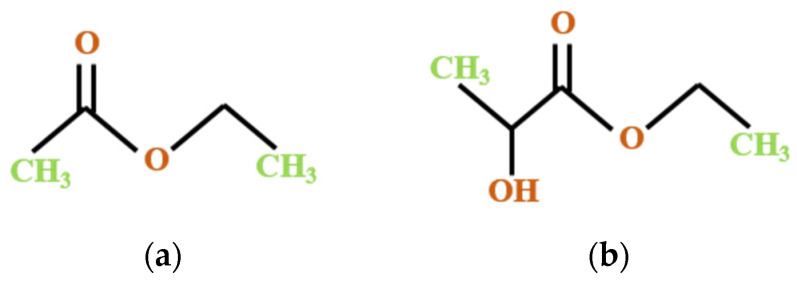
The chemical structure of green solvents used in the present study: (**a**) ethyl acetate; (**b**) ethyl lactate.

In relation to the principles of the bio-based economy, the conversion and valorization of recovered compounds into value-added products in a sustainable manner is of significant importance and constitutes the main scope of the circular economy action plan [22]. The design of new processes or the adaptation of existing technologies oriented toward the use of environmentally-friendly laboratory practices is the primary goal of the present study regarding carotenoid recovery from by-products and the upscale of these processes to the industrial level as future perspectives. In this regard, an alternative solution to the classic organic solvent extraction is offered by the green chemistry approach, and previous results indicate the use of green solvents such as ethyl lactate or ethyl acetate (Figure 2) to optimize lycopene extraction from waste materials [23,24].

Sustainable solvents employed for the extraction of carotenoids include the so-called “green solvents” and ionic liquids. Green solvents are generally produced from resources as biomass (wood, starch, vegetable oils, or fruits) or derived from non-toxic and/or biodegradable petrochemical-based solvents [25]. When used for extraction purposes, they should be able to penetrate into cell tissues, leading to high yields, short extraction times, cost-effectiveness, and sustainability, also preserving the biological activity of the extracted compounds.

Ethyl acetate belongs to the group of green solvents, which allows the extraction of lipophilic compounds in a sustainable way, having yields comparable to those of the extraction with hexane. Furthermore, it is considered as a low-toxicity solvent due to its rapid hydrolysis to ethanol and acetic acid during metabolism. It can replace benzene or methyl ethyl ketone and ethyl acetate/ethanol mixtures, and also act as a green alternative to dichloromethane in chromatographic separations [25].

Ethyl lactate is produced from the fermentation of carbohydrate feedstock, and it is entirely biodegradable in CO_2_ and water. Its miscibility with both hydrophilic and hydrophobic compounds makes it appropriate to extract a diverse range of metabolites additionally, it has the advantage of extracting both *trans*-and *cis*-lycopene isomers [26].

In 2005, the US Food and Drug Administration approved the use of ethyl lactate in food and pharmaceutical products, as a generally recognized as safe (GRAS) solvent, and earlier studies concerning sustainable chemistry and pharmacy concluded that ethyl lactate is a safe, efficient and potentially inexpensive solvent capable of extracting both polar and non-polar compounds from fruit and vegetable by-products [27].

Carotenoid extraction from agro-industry-derived wastes is a well-documented subject, and a growing body of scientific work can be found on this topic [5,6,25]. One of the most widely used techniques for carotenoid recovery from wastes and by-products is the classic organic solvent extraction, singly or in combination with other procedures, [7,28]; however, the vast majority of the used solvents in the process is originated from the petrochemical industry (which are highly flammable, corrosive, carcinogenic, and harmful substances). Furthermore, the major inconvenience of the classic solvent extraction is the elimination of the residual solvents to obtain a safe extract that can be integrated into food products [24].

According to a recent review about carotenoid extraction methods [23], ultrasound-assisted extraction (UAE) was found to be an efficient technique with regard to extraction time and temperature, as well as to the reduction in solvent consumption and the degradation and isomerization of lycopene [5,24].

Our study aimed to evaluate the total and individual carotenoid content, by high performance liquid chromatography (HPLC), found in tomato waste extracts, which were obtained by using green solvents such as ethyl acetate and ethyl lactate. The tomato by-products were freeze-dried and the extraction protocol was assisted by ultrasound. The recovered carotenoids were further used as functional ingredients in three different fatty acid profiled oils (flaxseed, grape seed, and hempseed oil) to examine how the incorporated bioactive compounds affect the oils’ rheological behavior.

## 2. Results and Discussion

### 2.1. Carotenoid Recovery from Tomato Processing By-Products by Ultrasound-Assisted Extraction (UAE)

In the present study, the extraction procedure of carotenoids from tomato processing by-products was assisted by ultrasound, as sound waves at frequencies greater than human hearing range can alter the materials both in physical and chemical ways by applying sponge effect and cavitation, which enhance solvent penetration in solid and facilitate the release of the target compounds [29]. It has been reported that ultra-sonication improves the lycopene content obtained from tomato by-products by 0.68 fold, whereas combined ultra-sonication with freeze-drying pretreatment enhances the lycopene yield up to 4.12 fold. These results might be also due to the disruption of tomato tissue, which implicitly improves the accessibility of the extraction solvent [30].

The spectrophotometric measurements of the extracts obtained by UAE using hexane, ethyl acetate (EA), ethyl lactate (EL), and a mixture of EA:EL (1:3 *v/v*) as solvents are presented in Table 1, and the results are expressed as mg carotenoids/g dry weight (dw).

The dry weight of the wet samples was calculated by determining their moisture content first, according to the official method No. 943.06 (Section 31.1.10B) of AOAC (Association of Official Agricultural Chemists), and expressed in percentage. The result showed a mean value of 18.17% dry weight. The freeze-dried samples were not subjected to this method as lyophilization involves the complete removal of water from the samples under vacuum, after they were frozen.

The results of the spectrophotometric measurements showed significantly higher total carotenoid content in the extracts obtained from the wet samples compared to the freeze-dried samples. Ethyl acetate proved to be with the highest efficiency in total carotenoid recovery, with 1.40 mg/g dw. Ethyl acetate has proven effect at extracting carotenoids from tomato waste in previous studies as well, but in combination with hexane [31].

Considering the lower accuracy of the spectrophotometric method and the consistent differences between samples in total carotenoid content, the extracts were subjected to HPLC analysis to identify and quantify individual carotenoids from the extracts.

### 2.2. Qualitative and Quantitative Analysis of the Recovered Carotenoids

**Figure 3:**
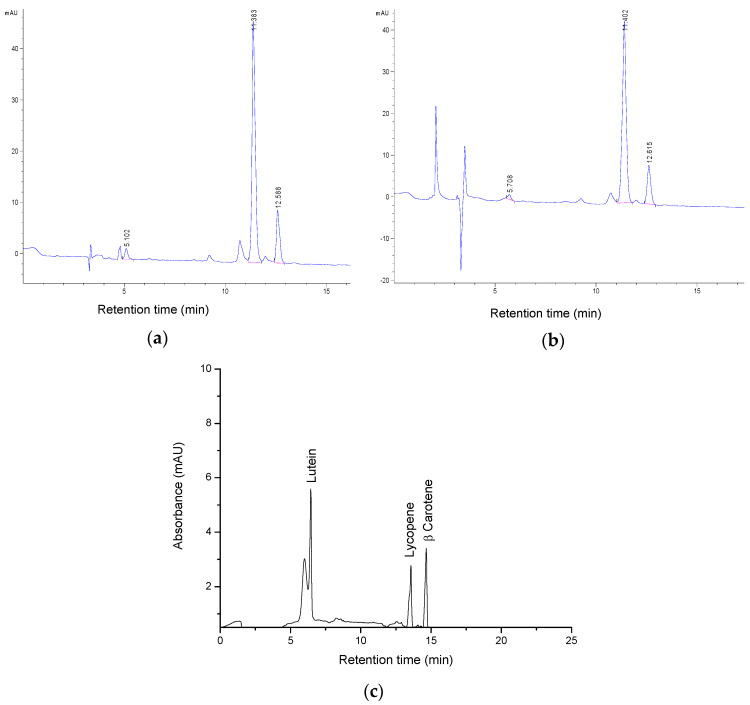
Chromatograms of the tomato by-product extracts using (**a**) ethyl acetate; (**b**) using ethyl lactate as solvent; (**c**) carotenoid standards: lutein R_t_ = 5.71 min; lycopene R_t_ = 11.40 min; β-carotene R_t_ = 12.62 min.

HPLC coupled to a diode array detector (DAD) identified individual carotenoids, precisely lycopene, β-carotene, and lutein of the samples and two of the most representative chromatograms are shown in Figure 3.

The results indicated lycopene as the primary bioactive compound in each extract, followed by β-carotene and lutein in smaller amounts. The quantification of individual carotenoids from the samples was calculated using the calibration curve of carotenoid standards (lutein, lycopene and β-carotene), and based on mass spectrometry measurements, and the results are presented in Table 2 as the mean values of three measurements +/− standard deviation.

In the case of freeze-dried samples, ethyl acetate was the most efficient solvent in the recovery of lycopene, with a 284.496 µg lycopene/g sample. The β-carotene content of the extracts showed the highest values when the combination of EA:EL was used, with 82.468 µg β-carotene/g sample and the lutein extraction was equally efficient with both EA and EA:EL, presenting similar results of 31.16 µg lutein/g sample. Previous studies conducted on lycopene and polyphenols content of three tomato cultivars subjected to different drying methods showed significant decrease in lycopene content in all cultivars when freeze drying was used [26]. The high rate of lycopene loss might be caused by oxidation given the exposure of the freeze-dried fibers to air and light.

Regarding the wet samples, the lycopene content and the β-carotene content of the extracts showed significantly higher quantities. When EL was used as a solvent, the lycopene content of the sections showed the highest amounts, with a 1324.89 µg lycopene/g sample. Concerning the β-carotene content of the extracts, EA was the most efficient solvent, resulting 235.57 µg β-carotene/g sample. The lutein content of the extracts obtained from wet samples was under detection limits regardless of the solvents used. The consistent decrease in carotenoid extractability from the wet samples when EA:EL was used as solvent might be caused by the differences in polarity of the two co-solvents which might affect the mechanisms involved in the extraction process.

The ability of lycopene to act as a potent antioxidant is believed to be responsible for protecting cells against oxidative damage and thereby decreasing the risk of chronic diseases. According to Saini et al. (2020), recent epidemiological studies revealed that the intake of tomatoes and blood lycopene levels are inversely associated with the risk of developing cancers at several anatomical sites, including the prostate, skin, stomach, breast, and lungs, and cardiovascular diseases [27]. Moreover, the consumption of tomatoes has been demonstrated clinically to have beneficial, protective effects against coronary artery disease and several neoplasms [27]. In addition, lycopene is particularly effective at quenching the destructive potential of reactive oxygen species [32].

Considering the significant differences in individual carotenoid content between freeze dried and wet samples it can be concluded that the drying method of tomato by-products should be deliberately selected for the targeted functional component (lycopene, β-carotene or lutein).

According to previous studies, the main factors influencing the extraction of carotenoids from tomato waste are temperature, time, and the nature of the solvent [31]. On the latter, ethyl lactate showed remarkable results in earlier experiments regarding carotenoid recovery from tomato waste, with yields ranging between 202.73 mg/kg at 25 °C and 243 mg/kg at 70 °C [33]. Concerning the elimination of the solvents in order to use a highly concentrated extract in the food industry, it has to be mentioned that EL is difficult to evaporate at temperatures that protect carotenoids from degradation; therefore, an alternative to its removal from the extracts could be freeze-drying, although this method seems to deteriorate carotenoids quality [26]. Ethyl acetate on the other hand can be evaporated at 35 °C at 150 mbar pressure, assuring concentrated carotenoid extracts, which can be further used as a natural colorant with antioxidant properties.

### 2.3. Color Change of the Oil Samples Enriched with Carotenoids, Extracted with EA

**Figure 4:**
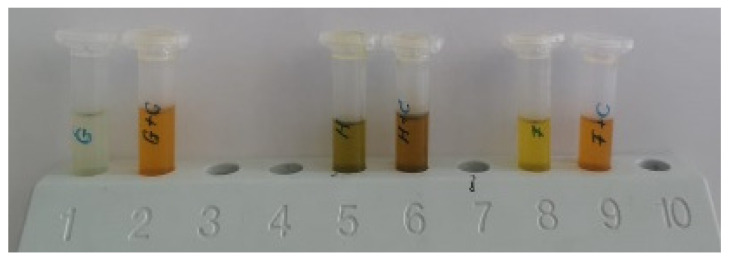
Grape seed oil, hemp seed oil and flaxseed oil enriched with carotenoids.

Due to their intense lipophilic nature, carotenoids integration into functional food products and their absorption mechanism in the body requires lipids [7]. According to the International Vitamin A Consultative Group (IVACG, 2017), carotenoids immersed in oil have a higher conversion efficiency to equivalent retinol (6-fold higher) when compared to carotenoids in vegetable matrices (e.g., 1 µg RE = 2 µg β-carotene in oil or 12 µg β-carotene in mixed foods). In this regard, we selected three types of oils, shown in Figure 4, with different fatty acid profiles, to incorporate the recovered carotenoids and to measure their flow behavior.

An intense color change can be observed in the oil samples added with carotenoids extract by increased redness and yellowness, indicating suitability as coloring agent. Additionally, previous studies confirm the reliability of carotenoids to increase the radical scavenging activity of oils, acting as antioxidant agents [34].

Vegetable oils hold an important place in the human nutrition as they represent a source of essential fatty acids, needed for the formation of cell membranes and the synthesis of hormone-like compounds called eicosanoids (e.g., prostaglandins, thromboxanes, and leukotrienes), which are significant regulators of blood pressure, blood clotting, and the immune response. Moreover, the ratio of the essential fatty acids linoleic acid (ω-6) and alpha-linoleic acid (ω-3) has been reported to play a fundamental role in reducing the incidence of chronic diseases, especially of cardiovascular character [35]. The ratio of w-6/w-3 between 2:1 and 3:1 was reported to be the optimal ratio that gives beneficial effects on human health [36].

The three oils enriched with carotenoids have different (ω-6)/(ω-3) ratios according to previous studies. Grape seed oil has a very high ratio (above 50:1), hemp seed oil had an optimal ratio of 3:1 and flaxseed oil had a ratio of 0.3:1 [37].

Hemp seed oil could become a sustainable alternative for other lipid typologies in the uprising vegetarian, vegan or gluten-free trends in diet, considering its constituents such as polyphenols, carotenoids, and tocopherols, all involved in antioxidant processes, which could play an important role in the protection of edible oils against lipid oxidation. To maintain their sensorial and nutritional properties, and to extend their shelf life, the oils are often added with synthetic antioxidants such as butylated hydroxyanisole, which might have harmful side effects according to recent studies [34]. Natural antioxidants such as carotenoids extracted from inexpensive biomass of the agricultural and industrial by-products could represent a sustainable, and healthier alternative to this process, optimizing human well-being at the same time.

As further evidence of the health benefits of carotenoids is discovered, there is likely to be an increased demand for the addition of carotenoids into functional food products. Based on a previous literature review about tomato processing wastes, however, there are several drawbacks regarding carotenoid behavior after extraction, which are related to their light sensitivity, hydrophobic nature, and bioavailability in the human organism after ingestion. More precisely, carotenoids need to be protected from light to exert their antioxidant properties.

The concept of functional foods relies on the ability of fortified or enriched foods to beneficially modulate one or more targeted functions in the body, by enhancing a certain physiological response and/or by reducing the risk of disease, beyond their nutritional effect [38]. Ingredient formulations based on carotenoids, however, need to be carefully engineered due to the multiple degradation mechanisms of carotenoids. Conventional emulsions, multi-layer emulsions, and solid-lipid particles are some of the suitable delivery systems in functional food formulations, as these systems offer multiple forms of carotenoid protection, while still permitting easy incorporation into foods [39].

### 2.4. Rheological Measurements of the Enriched Oil Samples

**Figure 5:**
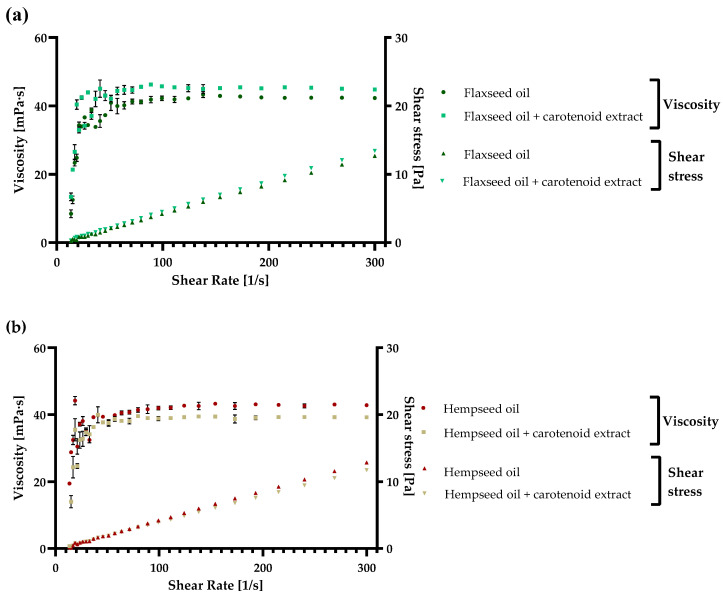

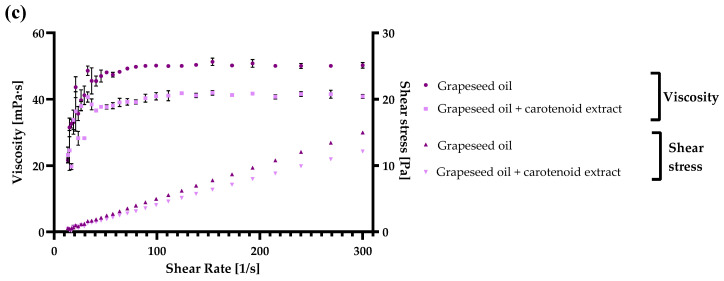
The viscoelastic behavior and shear stress of (**a**) flaxseed oil, (**b**) hempseed oil, (**c**) grapeseed oil, and their enriched variants with carotenoids, obtained with ethyl acetate extraction, were analyzed at 25 °C. Each value is displayed as the mean values of triplicate measurements ± SD (standard deviation); GraphPad Prism Version 8.0.1 (Graph Pad Software, Inc., San Diego, CA, USA).

Flaxseed, grape seed, and hempseed oils were used in the present study to describe the rheological, namely the flow behavior of the oils before and after carotenoid addition. To illustrate the viscoelastic features of three types of vegetable oils mainly used without thermal processing, they have been established together with their enrichment with a carotenoid extract. These oils are used in the food industry primarily in the production of healthy milk, salads, and yogurts, and they are also an essential element of the ketogenic diet [40,41]. Before the measurements, an equal concentration of carotenoids in each oil sample (40 µg/mL) was assured. The acquired results of the three oils (flaxseed, hempseed, and grapeseed) are presented in Figure 5a–c.

As observed in the figures, in both hempseed and grapeseed oil, when enriched with carotenoids, the viscosity decreased from 42.8 ± 0.1 mPa·s to 39.2 ± 0.2 mPa·s in the case of hempseed oil, and from 39.9 ± 0.3 mPa·s to 40.5 ± 0.0 mPa·s in the case of grapeseed oil. By contrast, in the case of flaxseed oil, the viscosity increased with carotenoid enrichment from 42.3 ± 0.1 mPa·s to 44.8 ± 0.0 mPa·s. Additionally, the shear stress of every sample increased logarithmically with the increase in shear rate reaching a final value between 11.69 and 15.06 Pa. Based on the viscosity and shear stress results, every analyzed sample with and without enrichment presented an ideal Newtonian behavior [8,42], and the final viscosity was in grapeseed < flaxseed < hempseed oil order.

In similar studies, the viscosity of grapeseed oils at 26 °C was 46.6 mPa·s, which is in accordance with our results, and at higher temperatures (50 °C), the viscosity decreased to 22.7 mPa·s. This decrease results from more significant thermal motion between the oil molecules, diminishing the intermolecular strength and consequently viscosity [43]. The viscosity of flaxseed oil, originating from oil-flax seeds was similar with our results with values ranging at 40 mPa·s at 25 °C, and the viscosity of fiber-flax seeds oil was above 45 mPa·s [44]. In hempseed oil, the viscosity ranged between 37.5 and 40 mPa·s, which was also in accordance with our results [45]. This study analyzed the integration of hempseed oil in different skin emulsions, due to various beneficial effects on the skin.

The introduction of carotenoid extract in an oil-based delivery structure presents an accessible route to enhance their bioavailability in foods [46]. Another essential delivery system that can also be considered is based on the encapsulation of these carotenoid enriched oils in maltodextrin and Arabic gum-supported wall material that can stabilize the emulsions [47]. As the nature of the oils is an important characteristic, and a decreased viscosity is critical, the oils used in the present study all present favorable features [48].

As concluded, the results obtained in the present article are in accordance with similar studies, and the analyzed viscosity between shear stress of 5 to 300 s^−1^ presented a viscosity between 40 and 50 mPa·s in the case of all three oils. These oils can all be suitably introduced into different food matrices based on the results. Further, the best and the lowest viscosity was obtained with hempseed and flaxseed oil based on edibility. Still, due to diminished viscosity in the case of enriched grapeseed oil, this substrate is also very suitable for consumption, especially after introducing the carotenoid extract.

## 3. Materials and Methods

### 3.1. Chemicals and Solvents

The solvents used for carotenoid extraction were of analytical grade; ethyl acetate (CAS:141-78-6), ethyl lactate (CAS:687-47-8), and hexane (CAS:110-54-3), as well as triethylamine and acetonitrile CHROMASOLV® (gradient grade, for HPLC, 99.9%), were purchased from Sigma-Aldrich, (Steinheim, Germany).

The lycopene, β-carotene and lutein standards were provided by BioMerieux (Marcy l’Etoile, France). Flaxseed oil, hemp seed oil, and grape seed oil were purchased from a local grocery store (Cluj-Napoca, Romania) and used without further purification.

### 3.2. Plant Material Used

The tomato by-products were obtained from the home processing of local tomato varieties. Briefly, the tomatoes were washed and cut into four pieces, then the juice was extracted with an electric tomato strainer (Vevor®). The resulting tomato pomace, containing tomato seeds, peels and small amounts of pulp, was kept at −20 °C until further analysis.

### 3.3. Determination of the Moisture Content of Samples

The moisture content of the samples was determined according to method No. 943.06 (Section 31.1.10B) of AOAC (Association of Official Agricultural Chemists). Briefly, 3 g of tomato by-products was weighed in a Petri dish and placed in a hot air oven for 48 h at 105 °C. The samples were transferred to a desiccator for 2 h, weighted, and the moisture content was calculated by the following equation:

(1) $$Moisture\;content\;\left(\%\right)=\frac{\left(W_{before}-W_{after}\right)}{W_{before}}\times 100$$

### 3.4. Pretreatment of the Samples (Lyophilization)

The biological material composed of frozen tomato by-products was lyophilized using a freeze-drier (Telstar LyoQuest table top freeze dryer, Terrassa, Spain) for 72 h. The chamber of the freeze drier was covered by aluminum foil to protect the samples from light.

### 3.5. Ultrasound-Assisted Extraction of Carotenoids

Carotenoids were extracted from wet samples and lyophilized samples using four solvents: hexane with a relative polarity of 0.009, ethyl acetate with a relative polarity of 0.228, ethyl lactate with a relative polarity of 0.800, and a mixture of EA:EL of 1:3 (*v/v*). The extraction procedure was assisted by ultrasound due to the advantages related to processing time and the possibility of using room temperature in the recovery of heat-sensitive bioactive compounds such as carotenoids, based on the protocol described earlier by Silva and coworkers [23,49], with slight modifications. Briefly, the samples (1 g) were finely ground in a mortar, placed in a Falcon tube, and 20 mL of solvent was added. The tubes were placed in an ultrasonic bath (Elma Schmidbauer GmbH, Singen, Germany) for 10 min at 35 °C and centrifuged at 10.8× *g* for 10 min at room temperature. The supernatant was collected, and the extraction procedure was repeated two more times with the same amount of solvent.

### 3.6. Spectrophotometric Measurements of the Extracts

An UV-VIS spectrophotometer (Perkin Elmer Precisely, Lambda 25, Waltham, MA, USA) measured the resulting extracts at a wavelength of A_450_. The absorbance of the blanks and the samples was also determined at 450 nm and total carotenoid content in each extract was calculated according to Equation (2) and the results were expressed as mg/g dry sample:

(2) $$Total\;carotenoids(\frac{mg}{g})\frac{(A\times V\times 1000)\times d}{2500\times w\times (1-m)}.$$

*A*: Determined absorbance,

*V*: Final volume of extract (mL),

*w*: Sample weight (g),

*m*: Moisture of the sample, and

2500: Molar extraction coefficient of carotenoids.

### 3.7. Qualitative and Quantitative Determinations of Carotenoids from the Extracts

Each extract was filtered through a 0.45 μm pore size nylon Millipore and injected into the HPLC system to detect individual carotenoids (lycopene, β-carotene, and lutein). An Agilent 1200 HPLC (Agilent Technologies, Santa Clara, CA, USA) was equipped with a diode array detector and with a high-purity reversed-phase EC 250/4.6 Nucleodur column (Macherey-Nagel, Düren, Germany). The mobile phases consisted of mixtures of acetonitrile: water (9:1, *v*/*v*) with 0.25% triethylamine (mobile phase A) and ethyl acetate with 0.25% triethylamine (mobile phase B), and the temperature was set to 25 °C as previously described [50,51]. The gradient started with 90% A at 0 min to 50% A at 10 min, and the percentage of A decreased from 50% at 10 min to 10% A at 20 min. The flow rate was set to 1 mL/min, and the chromatograms were monitored at 450 nm. HPLC peaks were identified using carotenoid standards (lycopene, β-carotene, and lutein), and quantification was based on the calibration curves of the standards.

### 3.8. Rheological Measurements of the Enriched Oil Samples

Prior to the integration of the carotenoids into the oil types, the extracts obtained by using EA were highly concentrated by evaporation of the solvent at 35 °C, 150 mbar and 180 rpm using a rotary evaporator (Heidolph rotary evaporator, Schwabach, Germany). The rheological analysis of the oil samples before and after the enrichment with carotenoids was performed based on previous studies [52,53], employing an Anton Paar MCR 72 rheometer (Anton Paar, Graz, Austria). The rheometer was provided with a Peltier plate-plate system (P-PTD 200/Air), with temperature regulation and a smooth parallel plate geometry of 50 mm diameter (PP-50−67300). To measure the samples (~3 mL), they were placed between the two plates, at a determined temperature of 25 °C, and a gap of 1 mm. The sample excess was withdrawn preceding the measurement, and the rest was left at rest for a duration of 10 min to ensure thermal equilibrium before measurements. The viscosity of the samples was assessed in triplicate at divergent and rising shear rates, with the speed of logarithmic stepwise sets between 5 and 300 s^−1^.

## 4. Conclusions

The recovery and reutilization of phytonutrients from agro-industry-derived by-products make the core of the circular economy action plan. Tomato processing by-products have a high content of bioactive compounds such as carotenoids, with extensive evidence regarding their health benefits in the human organism. Therefore, carotenoid extraction and their further revalorization in the food chain are of major interest to researchers and manufacturers. To obtain a safe extract that can be further integrated into functional food products, green solvents are necessary. The present study highlights the use of ethyl lactate and ethyl acetate in this sense.

The results showed that lyophilization of the by-products decreased the carotenoid content of the samples considerably. The main finding of this study is that ethyl lactate manifests a high performance in lycopene recovery from the wet samples (1324.89 µg/g DW), followed by ethyl acetate with insignificantly diminished values (1313.54 µg/g DW); however, ethyl acetate reveals the advantage of easy evaporation at 35 °C and 150 mbar, assuring a highly concentrated extract that can be easily incorporated into new food formulations. The oils enriched with carotenoids presented a decrease in viscosity of hemp seed and grapeseed oil, and an increase in viscosity of flaxseed oil, with a final viscosity between 40 and 50 mPa·s. Based on these results, it can be concluded that the carotenoid extract can improve the quality of oils, which ensures better suitability through their application in various food matrices.

Ethyl acetate in synergy with ultrasound assistance is an appropriate green system for carotenoid recovery from tomato processing by-products, which can significantly increase food safety. Nevertheless, further development is necessary to upscale the process to industrial levels.

## Figures and Tables

**Table 1:** Total carotenoid content of tomato processing by-product extracts based on spectrophotometric measurements at a wavelength of A450 nm absorbance, expressed as mg/g dw.

Solvent	Freeze-Dried Samples (mg/g dw)	Wet Samples (mg/g dw)
EA	0.23 ± 0.03	1.40 ± 0.03
EL	0.15 ± 0.02	1.07 ± 0.00
EA +EL	0.10 ± 0.01	0.50 ± 0.01
Hexane	0.11 ± 0.03	0.56 ± 0.02

**Table 2:** Individual and the sum of carotenoids identified in tomato by-product extracts determined by HPLC/DAD and expressed as µg/g dw.

Compound	λ_max_ (nm)	R_t_ (min)	Freeze-Dried Samples (µg/g dw)				Wet Samples (µg/g dw)			
			EA	EL	EA:EL	Hex	EA	EL	EA:EL	Hex
Lutein	425, 446, 476	5.71	17.18 ± 0.4	31.16 ± 0.7	31.16 ± 0.9	24.34 ± 0.6	n.d.	n.d.	n.d.	n.d.
Lycopene	448, 474, 508	11.40	284.50 ± 0.9	254.08 ± 1.1	270.67 ± 1.4	266.88 ± 1.4	1313.54 ± 1.9	1324.89 ± 2.0	690.28 ± 0.7	1184.15 ± 2.2
Β-Carotene	455, 480	12.62	73.58 ± 0.6	78.74 ± 0.8	82.47 ± 0.7	75.22 ± 06	235.57 ± 1.4	222.59 ± 1.6	122.20 ± 0.9	209.47 ± 1.3
Sum of carotenoids			375.26 ± 0.6	363.98 ± 0.8	384.3 ± 1.0	366.44 ± 1.2	1549.11 ± 1.7	1547.48 ± 1.8	812.48 ± 0.8	1393.62 ± 1.2

n.d. = not defined.

## Data Availability

Not applicable.
